# Relationships between Metabolic Comorbidities and Occurrence, Severity, and Outcomes in Patients with Acute Pancreatitis: A Narrative Review

**DOI:** 10.1155/2019/2645926

**Published:** 2019-10-07

**Authors:** Xu Li, Xiaolin Guo, Huifan Ji, Junqi Niu, Pujun Gao

**Affiliations:** Department of Hepatology, The First Hospital of Jilin University, Changchun, China

## Abstract

*Background*. The population of patients with acute pancreatitis treated by the staff at our department of gastroenterology includes those with mild and self-limited disease ranging to those with severe and fatal disease. Early diagnosis and accurate prediction of the severity and outcome of this disease, which is commonly seen by our department, is important for a successful outcome. Metabolic comorbidities (e.g., diabetes mellitus, fatty liver, obesity, and metabolic syndrome) are relevant to the severity and progression of many diseases. The objective of this review was to examine clinical relationships between metabolic comorbidities and occurrence, severity, and outcome of acute pancreatitis.

## 1. Introduction

Ten to twenty percent of patients with acute pancreatitis (AP) experience failure of multiple organs [1]; there is a high mortality rate in this group of patients. The trajectory of this inflammatory disease of the pancreas depends on severity. The 2012 revised Atlanta classification of pancreatitis divides the disease into the categories of mild, moderate, or severe. Severe AP has a significant risk of mortality (up to 40%), with the greatest mortality risk in patients with infected pancreatic necrosis [2]. Successful management of AP is challenged by the limited treatment options and the high mortality rates. Therefore, potential modifiable risk factors are still being investigated. The most common causes of AP are biliary obstruction (up to 60% of patients with AP) [3], excessive alcohol consumption (30%) [3–5], hyperlipidemia, and autoimmune disease [6].

The values for the prevalence of chronic metabolic comorbidities such as fatty liver (FL), diabetes mellitus (DM), and metabolic syndrome have increased worldwide. Interest in the roles of comorbidities in patients with acute and critical illness has developed over the past two decades [7–10]. In 2008, 35% of adults worldwide were overweight and 11% were obese [7]. Since 1990, the prevalence of DM has doubled worldwide; 285 million people had a diagnosis of DM in 2010 and up to 90% of these had type 2 DM in the past three decades [8, 11, 12]. Clinical understanding of metabolic syndrome has changed since it was first defined by Reaven [13] in 1988. It is a combination of factors that includes DM, hyperglycemia, dyslipidemia, arterial hypertension, and abdominal obesity [14].

Comorbidities have long been recognized as important determinants of severity and outcome. They are included in many classifications of disease severity (e.g., the acute physiology and chronic health evaluation (APACHE) II Score) [15]. Metabolic syndrome is associated with various diseases. However, the relationships between metabolic syndrome criteria and pancreatic diseases are not well-understood [16]. Only a limited number of articles examine the relationships between metabolic comorbidities and pancreatitis (Table 1).

The aim of this review was to examine the effects of FL, DM, and metabolic syndrome on the development, severity, and outcomes of AP.

## 2. Effects of Metabolic Syndrome

Metabolic syndrome is defined by the presence of a cluster of conditions that includes hyperglycemia, dyslipidemia, hypertension, and central obesity [9]. Investigation of the relationships between metabolic syndrome and the course of AP has been limited. One prospective cohort study of 140 patients in Saudi Arabia with a first attack of AP used the international diabetes federation definition of AP during investigation of the relationship between AP and metabolic syndrome [16]. They found a high prevalence (62.8%) of metabolic syndrome among patients with AP and a high correlation between metabolic syndrome and biliary pancreatitis in men and women [17]. However, they found that metabolic syndrome does not significantly affect the severity of AP [16]. Some of the individual elements of metabolic syndrome are associated with an increased risk and more severe course of AP. In contrast to the result reported by Sawalhi et al. [16], Mikolasevic et al. found that compared with populations of patients without metabolic syndrome, populations with metabolic syndrome have significantly greater values for incidence of moderately severe and severe AP [18]. Research on the effects of metabolic syndrome on AP outcomes using large, multi-center studies is needed.

### 2.1. Obesity

Taken together, study results indicate that the presence of obesity has negative effects on the trajectory of AP [16, 19–24]. Patients with obesity are at a greater risk for fatty infiltration of the pancreas. Results of clinical investigations indicate that obesity increases AP severity. This increased severity results from systemic injury of other organs or local pancreas-associated complications, or both; these injuries and complications result in increased mortality rates in patients with obesity and AP [25, 26].

Age and obesity have been identified by many studies as easily accessible and negative predictive factors in patients with AP [27–30]. An increased risk of early shock, pulmonary and kidney failure, and an extended hospital stay are associated with obesity [16, 19–24, 31]. De Waele et al. [32] found that in patients with AP, obesity is correlated with disease severity and an increased risk of local complications and mortality. Hong et al. [33] found that in addition to contributing to an increased risk of development of AP, obesity increases the risk of a poor prognosis in patients with AP. Sadr-Azodi et al. [20] study found that compared with individuals with a waist circumference between 75.1 and 85.0 cm, those with a waist circumference >105 cm had a statistically significant twofold increase in the overall risk of AP.

The body mass index (BMI) is a measure of obesity which is a BMI greater than or equal to 30 kg/m^2^ [34]. Some results of clinical studies and studies using animal models suggest that BMI is associated with an increased risk of more severe AP [35]. Researchers who examined a Swedish cohort of 179 AP cases found a nonsignificant positive trend between BMI and AP risk [36]. A study of a US cohort of elderly women with acute or chronic pancreatitis (660 cases) revealed a positive association between BMI and AP [37]. Using a multivariate analysis, Li et al. found that a higher BMI is independently associated with increased risks of acute kidney injury, acute respiratory distress syndrome, and deep venous thrombosis [38]. A meta-analysis (739 cases) performed by Martinez et al. [39] revealed that obesity (BMI ≥30 kg/m^2^) is a statistically significant risk factor for AP severity.

Emerging evidence suggests that an obesity paradox is present in patients with AP. Compared with patients with no obesity, patients with obesity and predicted severe AP, and no central adiposity, may experience better outcomes [40]. Therefore, obesity is not always an independent predictor for mortality and organ failure in patients with AP. Whether including obesity into scoring systems results in better prediction of severity remains to be determined.

The mechanisms via which obesity increases AP severity are unclear. One hypothesis assumes that chronic inflammation is associated with the adipose tissue, which releases pro-inflammatory cytokines. As a result, individuals with obesity are at a greater risk of lifestyle-related respiratory problems and chronic diseases [35, 41]. A second hypothesis is that the pancreas-associated low-level inflammation in patients with obesity increases and becomes an even greater inflammatory reaction when AP does occur. Inflammation of intra and peri-pancreatic fat tissue is an important part of AP pathophysiology. Because patients with obesity have greater amounts of pancreatic fat, the resulting inflammation is more severe.

A third mechanism that has been proposed is related to the FL that occurs in patients with obesity. FL negatively affects liver-associated detoxification of inflammatory mediators during AP. In a fourth possible mechanism, obesity is suggested to be associated with an increased risk of extra-pancreatic complications (e.g., kidney failure, shock, respiratory insufficiency, and a fatal outcome) [42, 43]. A fifth proposed mechanism is that obesity may be associated with an intensification of the immune response and subsequently more severe pancreatic injury. A severity scoring system (APACH-O) was proposed by Papachristou et al. [44], which includes obesity as an independent predictor of AP outcome. These researchers proposed that obesity is associated with an increase in AP severity via amplification of the immune response to injury. Finally, the intra abdominal pressure is typically increased in patients with obesity. This pressure increase results in a ventilation/perfusion mismatch of the lungs because the diaphragm is elevated. When a systemic inflammatory state such as AP is present, the effect will be even more severe and might result in reduced oxygenation of, and enhancement of an existing injury to, the pancreas [31].

### 2.2. Hyperlipidemia

After gallstones (up to 60%) [3] and alcohol (30%) [3–5], hyperlipidemia (including chylomicronemia) is the third most common cause of AP; hyperlipidemia accounts for 4–10% of cases of AP worldwide [45]. However, study results indicate that in China, hypertriglyceridemia is the second major cause of AP [46, 47]. The values for incidence were as much as 12.3% in 2003 [48], 18.1% in 2007 [49], and 25.6% in 2013 [46]. These values are much higher, compared with those in western countries. Severe hypertriglyceridemia accounts for 1–7% of all AP cases. In patients with primary or secondary hyperlipidemia, high circulating concentrations of triglycerides are associated with an increased risk of AP [45].

High triglyceride concentrations have been found in cases of AP with different etiologies. Therefore, hyperlipidemia is likely a primary precipitant of AP onset and an epiphenomenon [50, 51]. The adjusted hazard ratio for the association between serum triglyceride concentration and the overall risk of AP is 1.21 per 1 mmol/L triglyceride increment [52]. Kota et al. found that a diet that includes omega-3 fatty acids and the use of fibrates are the most effective treatments for patients with hyperlipidemia and AP [53]. Severe hypertriglyceridemia can also be treated using insulin and dextrose infusion, which can activate or cause the release of the lipoprotein lipase enzyme [54, 55]. The results of Anderson et al. [56] and Charlesworth et al. [51] studies indicated that a reduction of serum triglyceride concentrations to <5.65 mmol/L reduces abdominal pain in patients with AP and improves clinical outcomes.

Only a few studies have investigated the effects of hypertriglyceridemia on acute biliary pancreatitis outcomes. The presence of hypertriglyceridemia is associated with a poor prognosis [57, 58]. Zeng et al. study results suggested that high triglyceride concentrations are a risk factor for the development of severe AP and systemic and local complications in patients with AP [59].

No single mechanistic process has been found for the underlying pathogenesis of hypertriglyceridemic pancreatitis. Approximately one-fifth (15–20%) of patients with severe hypertriglyceridemia will develop AP [54, 60]. However, the etiology that initiates the disease process remains to be determined. Havel's theory [61] is the most commonly accepted pathogenic model. In this model, chylomicrons occlude pancreatic capillaries. The binding ability of albumin to aggregate into micelles with detergent properties is then overwhelmed by the hydrolysis of these triglyceride-rich lipoproteins, which releases high local concentrations of free fatty acids. The pancreatic vascular endothelium and acinar cells are subsequently injured by the free fatty acid micelle complexes. An acidic environment is created by the resulting ischemia, which induces increased free fatty acid toxicity. Cyclical micelle-free fatty acid production may partly contribute to the disease severity in patients with hyperlipidemic pancreatitis. Release and activation of pancreatic proteases and lipase and further autodigestion-associated injury are additional consequences of the acidic environment [57]. Pancreatitis would be induced in all cases with hyperchylomicronemia if Havel's proposed model was the only mechanism.

The hyperviscosity theory proposes that the presence of lipemia (i.e., high numbers of chylomicrons in the blood) contributes to the onset of hyperlipidemic pancreatitis [57]. Capillary blood flow in the pancreas is reduced by accumulation of chylomicrons in the microcirculation; ischemia then results. How hyperviscous blood induces selective pancreatic acinar cell ischemia remains unexplained.

Chang et al.'s [62] genetic theory identifies cystic fibrosis transmembrane conductance regulator mutation/variant/haplotype and a tumor necrosis factor promoter polymorphism as independent risk factors. Compared with patients without pancreatitis, a higher proportion of patients with hypertriglyceridemia and AP have this genetic polymorphism [62]. The e-4 allele of the *Apolipoprotein E* gene is more frequent in populations of patients with hyperlipidemic pancreatitis [63, 64]. Mitochondrial stress-induced direct tissue injury and lipotoxicity [62, 65, 66] can cause cytokine and inflammatory cascade up-regulation and increase the risk that the patient will have a systemic inflammatory response [65]. Hyperlipidemic pancreatitis is a complex disorder with an underlying mechanism that is likely affected by genetic, metabolic, environmental, and patient-specific factors.

## 3. Effects of Fatty Liver

The incidence of FL as a metabolic disease is increasing annually, and it is an increasing public health problem worldwide. The prevalence of FL is approximately 30% in developed countries and nearly 10% in developing countries. It is an important reason for an abnormal liver function test result [67]. Associations between FL and AP have been investigated [68, 69]. Patients with AP often also have FL because both diseases have the same contributing factors (e.g., hyperlipidemia, DM type 2, and obesity) [68–70].

Xu et al. assigned 2,671 patients with pancreatitis to an FL group or a nonFL group. Their study found that compared with nonFL group patients, FL group patients had a higher risk of death, and higher risks of severe AP and necrotizing AP. The values for incidence of local and systemic complications were also greater in the FL group. These results indicated that FL can affect severity and clinical outcome and may have prognostic value in patients with AP [68]. Mikolasevic et al. performed a retrospective analysis of a population of 822 patients hospitalized with AP. They found that the occurrence of moderately severe and severe AP and nonalcoholic FL disease (NAFLD) (odds ratio (OR) 2.13, 95% CI 1.236–3.689) were independently associated. Compared with a nonNAFLD group of patients, patients with NAFLD have a higher, but not statistically significant, death rate (5.6% vs. 4.3%; *p* = NS) [71]. Wu et al. examined the effect of NAFLD on AP severity. They found that compared with a nonNAFLD group, NAFLD has a clinically relevant effect on AP severity; the results suggested that the presence of NAFLD may be an early predictive factor for outcome in patients with AP [1]. Yoon et al. found that compared with patients with AP and without FL, patients with FL and AP had characteristics of more severe clinical disease (e.g., higher rates of local complications, persistent organ failure, and mortality). Even after adjusting for confounding variables (e.g., age, BMI, cause of AP) FL is a statistically significant risk factor for a severe disease course [70]. The results of a meta-analysis performed by Hou et al. to investigate the prognostic effect of FL on AP prognosis suggested that FL is independently associated with a severe clinical course of disease [72]. They also found a higher mortality in the FL-related AP group compared with the nonFL related AP group and a greater risk of systemic inflammatory response syndrome and local complications.

How FL increases the severity of pancreatitis remains to be determined. Populations of patients with FL often have greater rates of obesity [1] and hyperlipidemia. The pathogenesis via which obesity and hyperlipidemia lead to AP has been discussed above.

The inflammatory factor response easily results from the chronic inflammatory process that occurs in patients with obesity. As an inflammatory disease, FL also promotes chronic systemic inflammation [73–75] and likely exacerbates the severity of AP. The serum C-reactive protein level elevation that occurs in patients with FL suggests this chronic inflammatory process occurs [70, 76, 77], and a chronic pro-inflammatory condition in patients with FL may increase the severity of the course of AP [72].

A decrease in serum *Alpha-1-antitrypsin* levels may lead to the excessive activation of inflammation. *Alpha-1-antitrypsin* has significant anti-inflammatory properties because it affects a wide range of inflammatory cells (e.g., neutrophils, monocytes, macrophages, and mast cells). Results from rat and human AP models indicate that hepatic steatosis depresses *Alpha-1-antitrypsin* levels [78].

Kupffer cells are macrophages that reside in the liver. Approximately 70% of the liver's total macrophage numbers are Kupffer cells. They have an important role in AP pathogenesis via release of many different inflammatory factors [76]. During FL, Kupffer cells release of inflammatory factors is greatly increased. FL is also frequently accompanied by hyperlipidemia, which can induce accumulation of free radicals, disruption of the microcirculation, oxidative stress, and acinar necrosis in patients with AP [79–81].

The results of a recent study suggested that the *PPARα* signaling and fatty acid degradation pathways are involved in the pathological processes that occur in patients with AP and FL (APFL). This result suggests that FL can increase the severity of pancreatitis via these pathways [82]. NAFLD patients often have disorders that increase *Adipokine* (e.g., *CPR, IL-6, leptin*) levels and reduce *Adiponectin* levels. These changes increase the risk of systemic inflammatory response syndrome [77].

## 4. Effects of Diabetes Mellitus

Some studies have examined the effect of pre-existing DM on AP outcome [42, 83–89]. DM may affect the course of AP [87–89]. Pang et al. found that the risk of AP is approximately 30% greater in patients with diabetes. This finding is consistent with findings of five record linkage studies that included a total of 6,524 cases of AP [61, 90–96]. Using a large prospective cohort study, Nogaard et al. [89] found that DM is a statistically significant factor associated with mortality in patients with AP (*P* = 0.016). A large retrospective cohort study of nearly 57,000 patients with AP revealed that patients with DM have a higher risk of severe AP compared with matched counterparts with no DM (OR 1.21, 95% CI 1.16–1.26), a 58% higher risk of ICU admission, and a 30% greater risk of local complications [87]. However, the same study also found that despite this increased risk of severe AP, patients with DM and AP have a lower risk of mortality compared with those without DM (OR 0.77, 95% CI 0.65–0.91). This finding is similar to that of another retrospective cohort study, which found that although length of stay is significantly longer for patients with pre-existing DM than those without DM (median 5 vs. 4 d, *P* < 0.05), the results of a multivariate analysis indicated that severity of AP or in-hospital mortality is not significantly different between those with and without DM [88].

The details of the mechanisms via which DM affects outcomes in patients with AP have not been determined. It has been proposed that because patients with diabetes are likely to have a high comorbid burden (some of which is associated with AP etiologies such as hypertriglyceridemia and obesity), they will therefore have poorer reserves for a response to acute illness and an increased risk of severity and mortality [87, 88].

In summary, the studies that have investigated the effects of DM on outcomes in patients with AP have provided conflicting evidence. This is in part due to the study designs (retrospective cohort) used, which prevents the ability to isolate the effects of diabetes alone and control for confounding variables. Because the results from clinical studies are varied, more research is needed to determine the actual effects of DM on outcomes in patients with AP.

## 5. Conclusion

The findings from this review indicate that FL is associated with poorer outcomes in patients with AP, including an increased risk of severe AP, an elevated systemic inflammatory response, and an increased mortality risk. The distribution and degree of obesity is also important to consider when predicting the effect of obesity on outcomes in patients with AP. DM and metabolic syndrome and, especially, obesity and hyperlipidemia, also adversely affect the course of AP. More research is required, which should include isolation of the effects of other metabolic factors and controlling for the effects of confounding variables.

Evidence of the mechanisms underlying these associations is limited, and more work is required to determine the detailed mechanisms via which obesity, DM, metabolic syndrome, and other comorbidities are related and affect outcomes in patients with AP. This review identified possible mechanisms by which obesity and other metabolic comorbidities affect the inflammatory response during AP. Further investigation into the roles of *Adipokines* and *Cytokines* is warranted. Patients with one or more comorbidities will likely require acute care at some point. The presence of comorbidities will increase the complexity of care. Therefore, understanding how chronic comorbidities affect the course and outcomes of disease is extremely important due to the increasing prevalence of these comorbidities worldwide. Use of large-scale, well-designed studies to determine the effects of chronic comorbidities on clinically meaningful outcomes should be a research priority.

## Figures and Tables

**Table 1:** Clinical studies on the relationships between different metabolic factors and occurrence, severity, and outcome of acute pancreatitis.

Reference	Study design	Metabolic factors	Study conclusion
De Waele et al. [32]	Prospective study	Obesity	Body overweight and obesity represent a risk of more “severe” disease and the number and type of complications increase in categories of increasing BMI in acute biliary pancreatitis
Hong et al. [33]	Meta-analysis	Obesity	Obesity is not only associated with an increased risk of AP development, but it is also a poor prognostic factor for AP
Anderson et al. [56]	Retrospective observational study	Hyperlipdemia	A reduction of serum triglyceride concentrations to <5.65 mmol/L reduces abdominal pain in patients with AP and improves clinical outcomes
Xu et al. [68]	Retrospective cohort study	Fatty liver	Fatty liver could influence the severity and clinical outcome and may play a prognostic role in AP
Mikolasevic et al. [71]	Retrospective cohort study	Fatty liver	Presence of nonalcoholic fatty liver at admission can indicate a higher risk of developing more severe forms of acute pancreatitis and could be used as an additional prognostic tool
Nawaz et al. [88]	Retrospective cohort study	Diabetes mellitus	Diabetes did not have an effect on the course of AP
Shen et al. [87]	Retrospective cohort study	Diabetes mellitus	Diabetes may adversely affect the disease process of AP, it seems to protect patients from AP-related mortality

BMI = Body mass index, AP = Acute pancreatitis.

## Data Availability

The data used to support the findings of this study were supplied by Pujun Gao under license and so cannot be made freely available. Requests for access to these data should be made to Pujun Gao (gpj0411@163.com).
